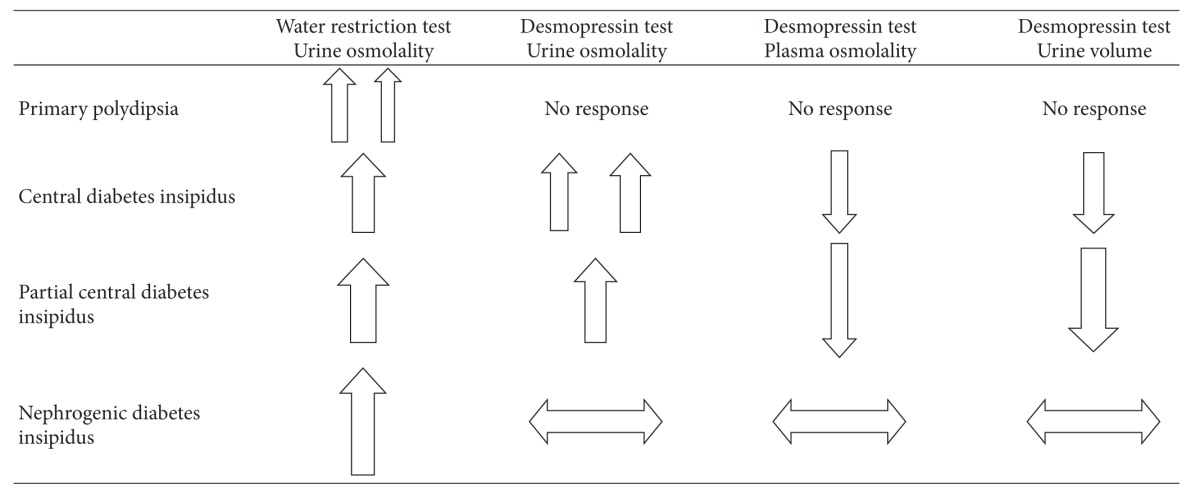# Diabetes Insipidus: A Challenging Diagnosis with New Drug Therapies

**DOI:** 10.5402/2013/797620

**Published:** 2013-03-24

**Authors:** Chadi Saifan, Rabih Nasr, Suchita Mehta, Pranab Sharma Acharya, Isera Perrera, Giovanni Faddoul, Nikhil Nalluri, Mayurakhan Kesavan, Yorg Azzi, Suzanne El-Sayegh

**Affiliations:** Division of Nephrology, Department of Medicine, Staten Island University Hospital, 475 Seaview Avenue, Staten Island, NY 10305, USA

## Abstract

Diabetes Insipidus (DI) is either due to deficient secretion of arginine vasopressin (central) or to tubular unresponsiveness (nephrogenic). Drug induced DI is a well-known entity with an extensive list of medications. Polyuria is generally defined as urine output exceeding 3 liters per day in adults. It is crucial to identify the cause of diabetes insipidus and to implement therapy as early as possible to prevent the electrolyte disturbances and the associated mortality and morbidity. It is very rare to have an idiosyncratic effect after a short use of a medication, and physicians should be aware of such a complication to avoid volume depletion. The diagnosis of diabetes insipidus is very challenging because it relies on laboratory values, urine output, and the physical examination of the patient. A high clinical suspicion of diabetes insipidus should be enough to initiate treatment. The complications related to DI are mostly related to the electrolyte imbalance that can affect the normal physiology of different organ systems.

## 1. Background

Though it is a rare disorder, diabetes insipidus was first described in the 18th century [1]. Diabetes insipidus (DI) is either due to deficient secretion of arginine vasopressin (AVP), also known as antidiuretic hormone (ADH) by the pituitary gland (central diabetes insipidus) or due to renal tubular unresponsiveness to AVP (nephrogenic DI). This leads to polyuria, polydipsia with hyposthenuria, causing dehydration and hypernatremia if the patient is deprived of water [2].

## 2. Etiology

Deficiency of AVP secretion is referred to as central DI, pituitary DI, or neurohypophyseal DI. About 50% of central DI cases are idiopathic [3]. It usually appears within 24 hours followed by a 2-3-week period of inappropriate antidiuresis. In a German study, only 8.7% of DI cases persisted for more than 3 months [4]. 

Close followup of patients diagnosed with idiopathic DI is necessary to detect slowly growing intracranial lesions. Lung and breast cancer are most common malignancies [5]. Leukemias and lymphomas are known to associate with DI [6]. 

The incidence of acute DI in severe head injury is high [7, 8]. 16% of central DI is secondary to head trauma. 

There are several autosomal recessive forms and X linked recessive forms were described causing DI. Gestational DI which manifests during pregnancy and usually remits several weeks after delivery is caused by deficiency of plasma AVP. [9].

Other causes of central DI include infiltrative disorders (histiocytosis X, sarcoidosis) anorexia nervosa, infections such as viral meningitis toxoplasmosis, inflammatory conditions including lupus erythematosis, wegener's, and vascular lesions, such as arteriovenous malformations or aneurysms (Table 1).

Majority of cases of hereditary nephrogenic DI have X-linked inheritance [10]. Hypercalcemia causes defective urinary concentrating ability which is generally reversible with correction of the hypercalcemia and may be associated with reductions both in sodium chloride reabsorption on the thick ascending limb of the loop of Henle, thereby interfering with the countercurrent mechanism. Persistent severe hypokalemia can have similar effects in the collecting tubule and the thick ascending limb of the loop of Henle. A variety of renal diseases can give rise to nephrogenic DI. Apart from lithium multiple medications are associated with nephrogenic DI (Table 2).

## 3. Epidemiology

Neurosurgical procedures, tumors, traumatic brain injury, tumors, infiltrative lesions, and malformations are the most frequent causes of DI.

**Normal Physiology of Vasopressin**

Arginine vasopressin is an antidiuretic hormone that is first synthesized in cell bodies of the nuclei in the hypothalamus and then transported to the posterior pituitary gland [11]. Although there are many factors responsible for the secretion of vasopressin like nausea, acute hypoglycemia, glucocorticoid deficiency, smoking, the most important stimulus is increased plasma osmolality [12]. The increase in plasma osmolality can be as small as 1%. [13]. The baroregulatory system usually does not cause the secretion of vasopressin during the normal circumstances unless there is a large volume loss, in which case there is release of some amount of this hormone [14]. Vasopressin acts as an antidiuretic by reabsorbing water via the principle cells of collecting ducts and the thick ascending loop of Henle, thereby increasing the plasma blood volume and decreasing the plasma osmolality [15, 16]. It can also cause contraction of the smooth muscles in the blood vessels [13] and release of von Willebrand factor [17].

**Vasopressin Receptors**

There are different types of receptor for vasopressin. The V1 receptor present in the endothelial cells leads to a pressor effect by the activation of Ca++ pathway whereas the V2R is the one responsible for water reabsorption by activating cyclic adenosine monophosphate (cAMP) in the kidneys and opening of the aquaporin channels [18, 19].

## 4. Pathophysiology: Figure 1

Irrespective of the cause of diabetes insipidus, whether central or nephrogenic, hereditary or acquired, the consequences are similar as shown in the chart above with loss of free water. This leads to dehydration if the patient is not able to keep up with urinary loss and may cause hypernatremia.

### 4.1. Central Diabetes Insipidus

The central causes of diabetes insipidus are either due to decreased secretion of vasopressin or secretion of partially functional form of vasopressin. The causes of central diabetes insipidus are hereditary, congenital or acquired. The hereditary form includes an autosomal dominant disorder with mutation of the AVP-neurophysin II gene [20]. One of the forms of the disease, the X-linked recessive and autosomal recessive forms due to mutations of the *WFS 1* gene responsible for Wolfram's syndrome can also cause diabetes insipidus [21]. Deficiency of vasopressin can occur during pregnancy from increased breakdown by an N-terminal aminopeptidase that is usually produced in the placenta [22]. There are many acquired causes of diabetes insipidus as described above.

### 4.2. Nephrogenic Diabetes Insipidus

The nephrogenic causes of diabetes insipidus are due to abnormalities of the vasopressin receptor V2R and aquaporin-2 protein water channels [23, 24]. The mechanism of lithium in causation of diabetes insipidus is still debated and there are many hypotheses [25]. Lithium causes decreased AQP2 and AQP3 expression and thus free water loss in the collecting duct [26]. Electrolyte abnormalities like hypokalemia and hypercalcemia can cause resistance to vasopressin [27, 28]. Chronic bilateral ureteral obstruction can cause decreased aquaporin protein expression [29, 30]. 

## 5. Clinical Manifestations

The age of presentation is dependent on the etiology, it can present at any age, and the prevalence is equal among males and females although there is one study showing higher prevalence in the males [31]. In an alert and conscious patient, diabetes insipidus presents with intense thirst (polydipsia), craving for ice water together with polyuria. The volume of fluid ingested may range from 2 L to even 20 L a day [32]. Less-severe cases may present with persistent enuresis. Most patients with an intact hypothalamic thirst centre maintain their fluid balance by drinking water. But patient who are unable to access free water as seen in neonates and elderly present with clinical features of hypernatremia and dehydration [33]. Lethargy, altered mental status, hyperreflexia, seizure, or, may be other presenting symptoms especially in the older age group, neonates and infants. Dehydration may lead to contraction of intravascular volume which in severe cases causes traction of dural veins and sinuses leading to intracranial hemorrhage. 

During pregnancy, diabetes insipidus is associated with oligohydramnios, preeclampsia, and even hepatic dysfunction [34].

## 6. Diagnosis of Diabetes Insipidus

Polyuria is generally defined as urine output exceeding 3 liters per day in adults. Usually polyuria could be caused by other conditions such as primary polydipsia, osmotic diuresis, and prostatic hypertrophy. It is crucial to identify the cause of diabetes insipidus and to implement therapy as early as possible to prevent the electrolyte disturbances and the associated morbidity and mortality.

In order to distinguish diabetes insipidus from other forms of polyuria, several blood tests have to be ordered including the blood glucose, plasma osmolality, bicarbonate levels, electrolytes, and urinalysis along with urine osmolality.

High blood glucose levels along with an osmolar excretion rate, which is equal to urine output multiplied by urine osmolality, above 1000 mosm/d indicates osmotic diuresis secondary to hyperglycemia. Other causes of osmotic diuresis could be due to urea as in post-AKI and mannitol and giving high intravenous sodium loads causing iatrogenic osmotic diuresis.

When the osmolar excretion rate is less than 1000 mOsm/d, two conditions have to be examined including primary polydipsia which is associated with a serum sodium of <140 meq/L and a dilute urine with urine osmolality of <100 mOsm/Kg on the one hand and diabetes insipidus on the other hand. It is associated with a serum sodium above 140 meq/L and a urine osmolality above 100 mOsm/Kg.

The water deprivation test helps to distinguish between the different causes of polyuria. It should be done by experienced physicians. It entails withholding any fluid intake from the patient. The normal physiologic response to water deprivation test leads to increase in antidiuretic hormone as the plasma osmolality increases and subsequently an increase in urine osmolality [35–37]. The effect of antidiuretic hormone would be maximal when the plasma osmolality reaches 295–300 mOsmol/Kg or when the serum sodium is above 145 meq/L. At this point, administering desmopressin will not further increase the urine osmolality only if we have deficient endogenous arginine vasopressin as in central diabetes insipidus.

The water restriction test helps to determine the cause of polyuria. First, it could be due to excessive drinking as in primary polydipsia. Second, it could be due to insufficient endogenous antidiuretic hormone, hence it is called central diabetes insipidus. And third, it could be due to resistance of the kidney to antidiuretic hormone, called nephrogenic diabetes insipidus.

Patients should stop drinking. After 2 to 3 hours of cessation of fluid intake, the urine volume and osmolality should be measured every hour and the plasma sodium and plasma osmolality every 2 hours. The test is continued till we reach one of the following endpoints.

1. The urine osmolality increases to reach a value above 600 mOsmol/Kg, it means that this is an appropriate response, and endogenous antidiuretic hormone is intact. The urine osmolality remains steady on 2 subsequent measurements and the plasma osmolality is rising. The plasma sodium level rises to reach a level above 145 meq/L or a plasma osmolality become between 295 and 300 mOsmol/Kg.
2. Desmopressin is then given in the last two conditions either subcutaneously or intravenously as 4 mcg or intranasally as 10 mcg. The urine volume and osmolality and plasma osmolality are followed closely and the variations should be recorded.
3. Different patterns to water restriction and desmopressin administration will help to discriminate between the different causes of polyuria [35, 38–40]. Refer to Table 3.
4. The urine osmolality increases in complete central diabetes insipidus with water deprivation test, usually to more than 300 mOsmol/Kg. Desmopressin leads to an increase in urine osmolality to more than 100% in complete central diabetes insipidus and 15–50% in partial central diabetes insipidus.

In nephrogenic diabetes insipidus, water deprivation test leads to a rise in urine osmolality but usually less than 300 mOsmol/Kg and desmopressin produces little or no change in urine osmolality.

In primary polydipsia, water deprivation test produces a rise in urine osmolality but desmopressin administration produces no change since the endogenous arginine vasopressin function and secretion are intact.

## 7. Differential Diagnosis

Diabetes insipidus must be differentiated from other causes of polyuria (Box 1).

## 8. Treatment

Mild cases of DI may not even need treatment and sufficient water intake may suffice. The removal of aggravating factors (e.g.,: reductions in glucocorticoidsthat directly cause free water clearance) improves polyuria.

**Central DI**

Earlier preparations available for treatment of central DI included acetone-dried extract from posterior pituitary of cowsand pigs.Variable duration of action and local irritation limited its use, and hencea more purified form of ADH preparation was made thatcould be administered intramuscularly. But side effects of abdominal cramps, angina and hypertension lead to the development of other drugs.

**Desmopressin**

DDAVP (1-deamino-8-arginine vasopressin): this has established itself as a drug of choice for the long-term management of central DI [41].It is a synthetic, long-acting vasopressin analog with minimal pressor activity but has nearly two-foldantidiuretic potency of arginine vasopressin. Pregnancy, and puerperium-associated DI will also benefit from desmopressin as it is resistant to degradation by circulating vasopressinase.Dosing is started at night to give relief for nocturnal polyuria, and day-time dosages are added as per need to control day-time symptoms. It can be given nasally, parenterally, or orally. Oral preparation is preferred for patients with sinusitis. Recently introduced lyophilisate melts under the tongue, may be preferred in children, and has a better bioavailability.

Starting dose of oral desmopressin is typically 0.05 mg twice daily and increased to a maximum of 0.4 mg every 8 hours if needed. Nasal preparation (100 mcg/mL solution) may be started at a dose of 0.05–0.1 mL every 12–24 hours through a metered dose nasal inhaler. Further dosing is individualized according to the degree of polyuria and is mainlyaimed at eliminating night symptoms. Patient should be monitored for water intoxication and hyponatremia. It has been linked todepression with an increased risk of suicide, agitation and erythromelalgia.

**Carbamazepine**

This anticonvulsant drug increasesthe renal sensitivity to the ADH effect [42]. In vivo studies showed carbamazepine decreased the urinary volume and increased the urinary osmolality by increasing that aquaporin 2expressionin the inner medullary collecting duct [43].

**Chlorpropamide**

This has beenfrequently usedin mild CDI. It potentiates the antidiuretic action of circulating arginine vasopressin and leads to a reduction ofurinary output bynearly fifty percent.It has an extensiveside effect profile includinghypo/hyperglycemia, hyponatremia, hyperlipidemia, hyperuricemia, hypercalcemia,hypokalemia, metabolic alkalosis, and myopathy. Hence certain studies proposed indapamide as a lowcost alternative for chlorpropamide.

**Indapamide**

This antihypertensive diuretic drug appears as an alternative drug for mild form of CDI as it increases urinary osmolality anddecrease serum osmolality [44].

**Nephrogenic DI**

The acute management of NDI involves correcting sodium levels by replacing free water deficits. Chronic NDI management involves reversing the cause if possible.

Lithum-induced NDI can be managed to a large extent by simplyincreasing water intake, given that the thirst mechanism is intact.If required,amilorideat doses 2.5–10 mg/dL can be used. Amiloridedecreases the lithium entry into principal cells by inhibiting ENaC. Thiazides induce hypovolemia and increase the proximal tubular water reabsorption and thus reduce polyuria. NSAIDS on the other hand can reduce the negative effect of intrarenal prostaglandins on urinary concentrating mechanism and help manage NDI. 

## 9. Prognosis

Central DI occurring after pituitary surgery usually remits within days to weeks but if structural damage has occurred to the stalk, it may even be permanent. The clinical course of chronic central DI is more of inconvenience to daily life than a dire medical condition. Currently available treatments do a good job to control symptoms but patients must be watched closely for side effects, water intoxication, and hypernatremia.

## Figures and Tables

**Figure 1:**
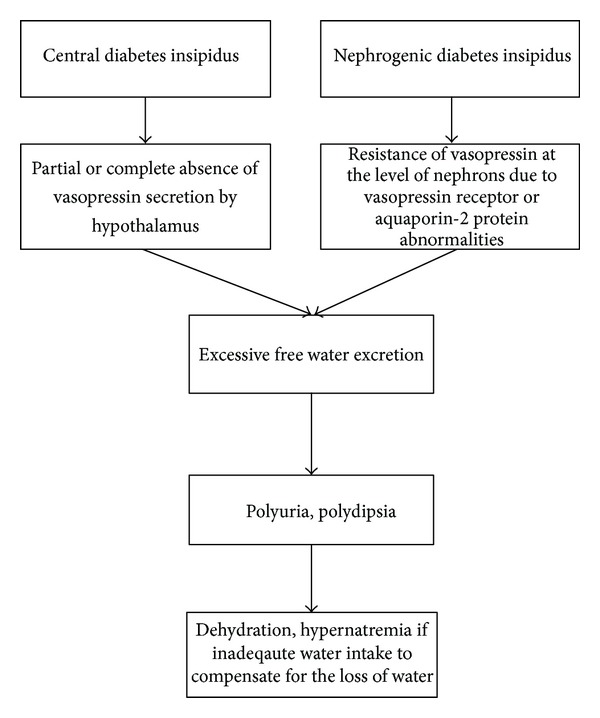


**Box 1:**
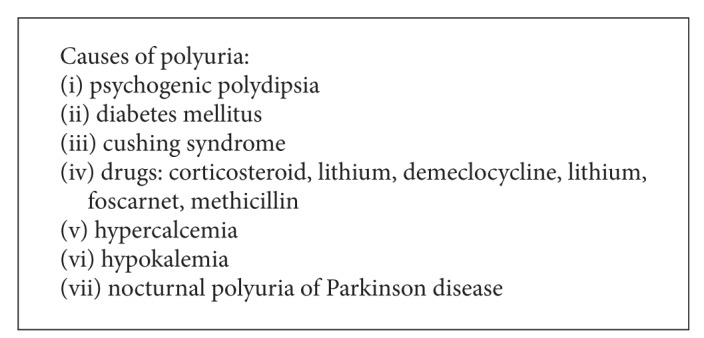


**Table 1:** Causes of central diabetes insipidus.

Postsurgery: transfrontal/transsphenoidal
Traumatic brain injury
Tumors
Primary
Craniopharyngioma
Hypothalamic tumors (glioma)
Pitiutory adenoma
Dysgerminoma
Meningioma
Hematologic
Lymphoma, leukemia
Metastatic
Breast, lung
Infections
TB meningitis
Viral meningitis
Cerebral abscess
Toxoplasmosis
Granulomas
Sarcoidosis
Histiocytosis
Inflammatory
Systemic lupus erythematosus
Scleroderma
Wegener's disease
Vascular
Aneurysm
Hypoxic encephalopathy
Sheehan's syndrome
Chemical toxins
Snake venom
Tetrodotoxin
Genetic

**Table 2:** Causes of nephrogenic diabetes insipidus.

Drugs
Lithium
Ofloxacin
Demeclocycline
Amphotericin B
Aminoglycosides
Cisplatin
Cidofovir
Foscarnet
Ifosfamide
Didanosine
Ifosfamide
Postobstructive
Vascular
Sickle cell disease and trait
Acute tubular necrosis
Metabolic
Severe hypercalcemia
Severe hypokalemia
Infiltration
Amyloidosis
Sjögren's syndrome
Granulomas
Sarcoma
Granulomas
Sarcoidosis
Genetic

**Table 3:**